# Divisive feedback can underlie phasic firing but is precise coincidence detection adequately robust?

**DOI:** 10.1186/1471-2202-13-S1-P60

**Published:** 2012-07-16

**Authors:** Xiangying Meng, Gemma Huguet, John Rinzel

**Affiliations:** 1 Biology Department, University of Maryland, USA; 2 Courant Institute of Mathematical Sciences, New York University, New York, NY, 10012, USA; 3 Center for Neural Science, New York University, New York, NY, 10003, USA

## 

Some neurons fire only phasically, say, once or a few times, at the onset of an adequate depolarizing current step but not for steady or slow inputs. This property of phasic firing is known as Type III excitability. The underlying mechanism for Type III behavior is a dynamic, voltage-gated, negative feedback that can be recruited subthreshold, preventing the neuron from reaching spike threshold if the voltage/input rises not fast enough. If the negative feedback is fast, say, comparable to the membrane time constant phasic neurons can show extraordinary temporal precision for phase locking and coincidence detection. Exemplars are found in the auditory brain stem where precise timing is important for sound localization and where interaural time differences are computed by coincidence detection with tens of microseconds resolution.

We have carried out a comparative study with models that can be cast in both divisive and subtractive mechanisms to gain insight into the dynamical mechanism for the potentially high temporal precision of Type III neurons. Our control case is a widely-used, conductance-based, 8-variable phasic model [1] for auditory neurons for which a low-threshold (Kv1.1) potassium current (IKLT) is the primary fast subthreshold negative feedback mechanism (subtractive dominant mechanism). This RM03 model loses its phasic properties if the conductance of IKLT is frozen at its resting value. However, we can rescue the Type III excitability with a divisive negative feedback mechanism by modifying the inactivation gating for sodium current -- by left-shifting the V-dependence of steady-state inactivation.

We have developed reduced 2-variable models, both divisive and subtractive dominant versions, that mimic semi-quantitatively and qualitatively the behaviors of the control, RM03. Our reduced models permit phase-plane analysis and thereby prediction and insight into the phasic firing properties. For the reduced models we identify and interpret geometrically distinguishing features, such as operating point and temporal integration window. We find that subtractive spikers can out-shine divisive spikers for temporal precision and coincidence detection, especially as the mean input level increases. Increased mean depolarization can move the operating point outside the useful range for the subthreshold mechanisms. The subtractive dominant mechanism can prevent excessive depolarization by directly counteracting increased input. But mean depolarization is a major problem in the divisive case because depolarization leads to loss of excitability. Here, synaptic inhibition can rescue excitability and improve temporal precision. We demonstrate these properties by comparison of tuning curves for coincidence detection (firing rate vs phase-difference for rectified sine wave input). With increased stimulus intensity the subtractive mechanism loses sensitivity because firing rate increases in the anti-phase troughs of the tuning curves (increased false positives); similarly for the divisive mechanism before excitability deteriorates (from excessive depolarization). Remarkably, when both mechanisms are combined the firing in the anti-phase troughs is significantly reduced and the tuning curves are more narrowly tuned. Our characterizations for this collection of models suggest qualitative aspects of Type III excitability that should apply to many neuron models and to phasic neuronal network firing rate models.
